# Robotically controlled ablation for atrial fibrillation: the first real-world experience in Africa with the Hansen robotic system

**DOI:** 10.5830/CVJA-2012-015

**Published:** 2012-06

**Authors:** Faizel Lorgat, Evan Pudney, Helena Van Deventer, Sam Chitsaz

**Affiliations:** Department of Cardiology, Christiaan Barnard Memorial Hospital, Cape Town, South Africa; Department of Cardiology, Christiaan Barnard Memorial Hospital, Cape Town, South Africa; Department of Cardiology, Christiaan Barnard Memorial Hospital, Cape Town, South Africa; Cardiac Biomechanics Laboratory, University of California San Francisco, San Francisco, California, USA

**Keywords:** atrial fibrillation, catheter ablation, atrial flutter, robotic navigation, computer-assisted ablation

## Abstract

**Background:**

We report the first single-centre experience in Africa with the Sensei X robotic navigation system in an unselected subset of patients with atrial fibrillation (AF).

**Methods:**

Data were recorded prospectively of all consecutive patients who underwent robotically assisted catheter ablation therapy using the Sensei X robotic navigation system at the Christiaan Barnard Memorial Hospital, Cape Town, South Africa, from July 2009 to July 2010. Outcomes were defined at one and nine months.

**Results:**

A total of 95 patients were included: 63% had only AF and 37% had AF plus atrial flutter. AF was of the persistent type in 81% of patients. The mean procedure, fluoroscopy and ablation times were 220.6 ± 89.6 min, 31.0 ± 20.4 min, and 61.3 ± 28.1 min, respectively. Both fluoroscopy and procedure times were significantly longer for the first 19 patients compared with the remaining 76 patients (43.5 ± 22.7 vs 27.8 ± 18.5 min and 274.7 ± 90.2 vs 207.1 ± 84.7 min, respectively, *p* = 0.002). The procedural endpoint of the study was successfully achieved in all patients. After one attempt, 27% were discharged from hospital off anti-arrhythmic drugs (AADs). At a median of nine months’ follow up, 74% were AF-free off AADs, and 11% were AF-free on AADs, yielding a total freedom from AF of 84% without any redo procedures. Freedom from relapse after 1.12 procedures was 88%.

**Conclusion:**

The Sensei X™ robotic navigation system offers a safe and effective approach for the treatment of AF. There was a learning curve with regard to fluoroscopy and procedure time, after which point reduction in radiation exposure and operator strain, as well as improvement in procedure throughputs were even more pronounced.

## Abstract

Atrial fibrillation (AF) is the most common sustained cardiac rhythm disturbance in the general population.^1^ As life expectancy and average age increase, it is estimated that the number of patients affected by AF will increase 2.5-fold over the next five decades.^2^ Uncontrolled AF may result in devastating complications such as haemodynamic impairment and increased risk of stroke, which in turn have a dramatic impact on quality of life, morbidity and mortality. Hence, it is imperative to advance treatment options available for patients suffering from this condition.

Over the past decade, catheter ablation has been proven to be effective in treating various types of arrhythmias. According to the latest guidelines, catheter ablation is indicated in cases of symptomatic arrhythmias refractory to conventional anti-arrhythmia therapies.^3^-^6^ Specifically, limited success of anti-arrhythmia drugs in the treatment of AF has made this condition the dominant indication for catheter ablation in high-volume electrophysiology centres.^1^

So far, two catheter-based approaches have emerged as accepted strategies for AF treatment: (1) ostial segmental disconnection of all pulmonary veins from the adjacent atrial tissue, and (2) circumferential pulmonary vein ablation (CPVA).^6^ The circumferential approach was found to be significantly more effective than segmental ablation for paroxysmal AF. However, these two seemingly different strategies are converging towards a unified strategy (i.e. circumferential approach) and are reporting similar success rates.^7^ Regardless of strategy, however, the safety and efficacy of these strategies and others under investigation are highly operator dependent and require reproducible catheter movements and optimal catheter stability and contact during mapping and ablation energy delivery.

In recent years, remote catheter navigation systems have been introduced to improve precision during catheter manipulation, reduce physical demands on the operator, minimise fluoroscopy time, and increase patients’ safety by avoiding serious complications.^8^ The Sensei X robotic navigation system (Hansen Medical, Mountain View, CA) and the Niobe magnetic navigation system (Stereotaxis, St Louis, MO) are two commercially available remote catheter navigation systems currently available on the market.

Performance of the Sensei X system has been evaluated in American and European studies.^9^-^12^ Researchers investigated technical characteristics and outcomes of robotic AF ablation and found that the complications and recurrence rates with the robotic system were comparable to those of manual ablation,^13^ while the amount of radiation exposure was significantly lower with the robotic navigation.^14^ The learning curve in early cases resulted in longer procedure times at each centre, mostly due to lack of experience with the set-up, which gradually improved.^15^

In this article, we report the first real-world experience in Africa with the Sensei X robotic navigation system in an unselected subset of patients with predominantly persistent AF.

## Methods

This was a prospective, single-centre, single-operator study performed at the Christiaan Barnard Memorial Hospital, Cape Town, South Africa. The data of all consecutive symptomatic patients with AF who underwent robotically assisted catheter ablation therapy using the Sensei X robotic navigation system (Hansen Medical, Mountain View, CA) from July 2009 (the time of acquisition of the robotic system) to July 2010 were recorded in an electronic database.

The exclusion criteria were: (1) patients with cardiac dysrhythmia without documented AF, e.g. only atrial flutter (AFlut) or regular supraventricular tachycardia (SVT), (2) congestive heart failure with New York Heart Association class IV or ejection fraction of < 30%, (3) recent acute coronary syndrome within two weeks, (4) impaired mental status that may affect timely taking of anti-arrhythmia drugs, (5) severe cerebrovascular disease, and (6) patient’s decline to sign informed consent. Ninety-five patients were finally included. Institutional research board approval was waived for this study.

AF types were defined based on the latest European Society of Cardiology (ESC) guidelines^5^ as follows: paroxysmal AF is a self-terminating episode that lasts up to seven days; persistent AF either lasts between seven days and one year, or requires termination by cardioversion with drugs or direct current; long-standing persistent AF lasts a year or more.

Screening (fluoroscopy) time involved the total minutes the X-ray was applied by the operator to visualise the position of devices or set up the system in the patient, using single-plane pulsed fluoroscopy with a rate of seven frames per second, i.e. the time needed to place the diagnostic catheter in the coronary sinus, to perform a trans-septal puncture, to assist with the initial creation of a three-dimensional (3D) map, and occasionally thereafter to verify accuracy of 3D mapping. Ablation (radiofrequency) time was defined as the total minutes the operator used electrical current to ablate the rebel atrial foci. The procedure time was defined as the time between first venipuncture in the operating room and full recovery of the patient from anaesthesia.

All high-risk patients (CHADS_2_ score ≥ 2 or patients with impaired left ventricular function) and all patients with long-standing persistent AF were anticoagulated on warfarin for four weeks until five to seven days prior to the procedure. Subcutaneous enoxaparin (1 mg/kg daily) was administered three to four days pre-procedure. Low-risk patients (CHADS_2_ score < 2) who had persistent AF by definition but who were in sinus rhythm prior to the procedure because of drugs or recent direct-current cardioversion were not always anticoagulated on warfarin. All patients were instructed not to ingest any solid foods within six hours prior to their procedure.

Three-dimensional mapping was performed to facilitate robotically assisted catheter ablation. In all patients, 3D reconstruction of the corresponding atrial chamber anatomy was performed with either the EnSite NavX system (76 patients) or CARTO electro-anatomic mapping system (19 patients).

## Procedure

All procedures were performed under general anaesthesia using propofol and remifentanil. All patients received a bolus of dexamethasone as part of the anesthesia to reduce the incidence of postoperative nausea. The use of corticosteroids may also be effective for preventing post-ablation recurrence of atrial tachycardia.^16^

A temperature probe was routinely placed into the oesophagus for continuous intra-oesophageal monitoring during radio-frequency (RF) ablation. A multipolar catheter was placed into the coronary sinus via a 7F sheath in the right femoral vein. An ablation catheter, either a 3.5-mm ThermoCool® catheter (Biosense-Webster, Diamond Bar, CA, USA) or a Cool Path™ Duo RF ablation catheter (St Jude Medical, St Paul, MN, USA) was placed into the Artisan sheath (Hansen Medical, Mountain View, CA). The steerable sheath system (SSS) was inserted via a 14F sheath in the right femoral vein and advanced manually into the right atrium under fluoroscopic visualisation.

Approximately 0.5 cm of the ablation catheter was exposed. After placement of the SSS in the inferior right atrium was confirmed, the position of the SSS was registered into the robotic catheter remote-control system. This registration involved the use of LAO 30 and RAO 30 fluoroscopic views of the heart (anterior–posterior and lateral) to allow localisation of the SSS in the 3D space.

In all patients, one trans-septal puncture was performed under fluoroscopic guidance. In exceptional patients trans-oesophageal echocardiographic guidance was used to facilitate trans-septal puncture. The trans-septal puncture was performed manually. Through this trans-septal puncture, a long sheath was advanced into the left atrium, and a circular mapping catheter was introduced into the left atrium. The ablation catheter was robotically guided into the left atrium through the same transseptal puncture site (adjacent to the shaft of the circular mapping catheter). This catheter was used for ablation.

Systemic anticoagulation was initiated after the first trans-septal puncture with the use of intravenous unfractionated heparin with a target activated clotting time (ACT) of approximately 250 s. A 15–25 variable loop Biosense LASSO circular mapping catheter (Biosense-Webster, Diamond Bar, CA, USA) was used for mapping the pulmonary vein (PV) antrum. With this configuration, the final set up before initiation of ablation included the following: a robotically controlled steerable sheath housing the ablation catheter (this is performed by a physician at the console) and a manually controlled circular mapping catheter handled and moved by the same physician at the procedure tableside.

When intra-oesophageal temperature rises were noted, power output was reduced to 20 W. At the end of the procedure, systemic anticoagulation was discontinued and occasionally partially reversed with intravenous protamine before removal of the vascular sheaths

## Ablation

Ablation along the pulmonary vein antrum, which encompasses the posterior wall, septal aspect of the right-sided pulmonary veins, and the ridge between the left atrial appendage and left pulmonary veins, was performed with the Artisan robotic system until disappearance of local PV potential, with the endpoint of electrical isolation of all PV antra. Radiofrequency power was set at 30 W with a maximum temperature limit of 45°C with irrigation using a heparinised saline infusion (2 000 IU/l) at a rate of 17 ml/min via the Cool Flow pump (Biosense-Webster, Diamond Bar, California, USA).

After PV isolation was conducted, the circular ablation catheter was withdrawn. If AF persisted after PV isolation, flecainide was administered intravenously up to a maximum of 150 mg unless contraindicated, in which case amiodarone up to a maximum dosage of 300 mg was infused. If this restored sinus rhythm then no further left atrial ablation was performed. If AF persisted, then mapping for complex fractionated atrial electrograms was performed. If complex fractionation was noted along the coronary sinus musculature, then ablation was performed along the endocardial aspect of the coronary sinus. If AF organised into a regular tachycardia at any stage during mapping and ablation, then a detailed activation map was constructed and the appropriate ablation was performed to terminate the flutter or focal atrial tachycardia. If AF persisted despite all of these efforts, a direct-current cardioversion was performed.

Ablation along the posterior cavo-tricuspid isthmus was performed in 30 patients with the robotic system in conjunction with the 3.5-mm Navistar® ThermoCool® catheters or Cool Path Duo. Bidirectional block was confirmed with differential pacing techniques (proximal coronary sinus/postero-lateral right atrial pacing). All patients received enoxaparin (Clexane) 40 mg bd a few hours post procedure until discharge the next day (two doses on average).

## Follow up

The procedural endpoint of the study was whether isolation of all PV or SVC potential foci was successfully achieved, or conversion to manual catheter ablation was needed. Our primary endpoints were recurrence of symptomatic AF lasting more than one minute at nine months post procedure, major cardiac complications that needed intervention/surgery, and all-cause mortality. A two-month blanking period was defined in which episodes of AF occurring within that time period were not considered recurrence.

Follow up was scheduled at one and six months. A resting ECG was performed in each follow-up visit. Outside of the scheduled follow ups, additional assessment or investigation was considered if symptoms warranted it. Patients previously on warfarin resumed oral anticoagulation therapy with warfarin the day after the procedure, and oral anticoagulation therapy was continued for at least one month for patients with paroxysmal AF.

Patients with persistent and long-standing persistent AF received a recommendation to take warfarin for at least three months, after which warfarin was discontinued if sinus rhythm was maintained and the CHADS_2_ score was less than 2. Warfarin-naive patients were commenced on aspirin 81 mg and Plavix 75 mg for one month only.

All of these patients were advised to take aspirin indefinitely. Antiarrhythmic drugs were continued for a one- to three-month period. All patients received esomeprazole (Nexium) 40 mg daily for one month.

## Statistical analysis

Categorical and continuous variables are presented as frequency (percentage) and mean ± one standard deviation, respectively. Distribution of the continuous variables was assessed using the Kolmogorov-Smirnov test. To compare normally distributed continuous parameters, the independent samples Student’s *t*-test was performed, while the Mann-Whitney test was used to compare non-normally distributed variables. Categorical parameters were compared using the Fisher’s exact or χ^2^ test. Statistical Package for the Social Sciences (SPSS) v 15.0 was used for plotting the graphs and statistical analyses and tests. Differences were considered significant at *p* < 0.05.

## Results

A total of 100 procedures were performed in the defined period. Five robotic ablations were redo procedures on patients who had previously undergone robotic ablation in our centre. Ninety-five patients met all the selection criteria and were enrolled in the final analysis. Demographics and baseline clinical characteristics are summarised in Table 1. Fourteen patients (15%) were receiving more than one AAD pre-procedure. For eight patients (8%), manual ablation was tried previously at least once (i.e. the robotic procedure was a redo ablation).

**Table 1.** Baseline Characteristics Of All Enrolled Patients (*n* = 95)

Male, *n* (%)	71 (75)
Age (years)	59.4 ± 9.9
AF duration (years)	5.3 ± 6.1
Ischaemic heart disease, *n* (%)	23 (24)
Diabetes mellitus, *n* (%)	9 (9)
Systemic hypertension, *n* (%)	49 (52)
Hypercholesterolaemia, *n* (%)	38 (40)
Structural heart disease, *n* (%)	11 (12)
Pre-procedure anti-arrhythmic drugs, *n* (%)	
Sotalol	19 (20)
Amiodarone	27 (28)
Flecainide	10 (11)
Dronedarone	1 (1)
Propafenone	1 (1)
Sotalol + Flecainide	1 (1)
Beta-blockers	34 (36)
Pre-procedure digitalis, *n* (%)	4 (4)
Pre-procedure warfarin, *n* (%)	36 (38)
Ejection fraction (%)	61.3 ± 8.2
Left atrial size (cm)	4.3 ± 0.6
Procedure time (min)	220.6 ± 89.6
Screening time (fluoroscopy time, min)	31.0 ± 20.4
Ablation time (min)	61.3 ± 28.1

Sixty patients (63%) had only AF, and 35 patients (37%) had documented atrial flutter (AFlut) along with their AF. In the majority of patients (81%), AF was of the persistent type. In patients who had AF + AFlut, AFlut was mainly typical (78%). Different types of AF and AFlut in the study population are summarised in Table 2.

**Table 2.** Types Of Dysrhythmia In The Study Population

*Dysrhythmia*	*n (%)*
Atrial fibrillation	95 (100)
paroxysmal	13 (14)
persistent	77 (81)
long-standing persistent	5 (5)
Atrial flutter	35 (37)
typical	29 (31)
atypical	2 (2)
left atrial	3 (3)
peri-mitral	1 (1)

The procedural endpoint was achieved in all 95 patients enrolled in the study. All ablation procedures were performed using the Sensei X robotic navigation system. Seven patients (7%) needed medical cardioversion during the ablation procedure, three (3%) needed electrical cardioversion, and nine patients (9%) needed both.

**Fig. 1.**
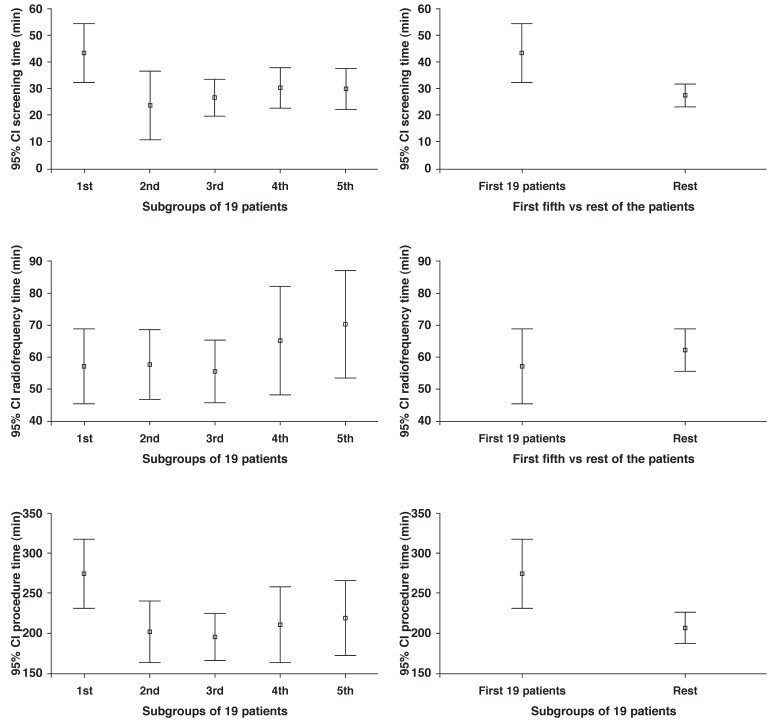
Error bars representing screening time, radiofrequency time and procedure time for subgroups of 19 patients; CI, confidence interval.

The mean procedure, fluoroscopy and ablation times were 220.6 ± 89.6 min, 31.0 ± 20.4 min, and 61.3 ± 28.1 min, respectively. Both fluoroscopy (screening) and procedure times were significantly longer for the first 19 patients compared with the remaining 76 patients (43.5 ± 22.7 vs 27.8 ± 18.5 min, *p* = 0.002 and 274.7 ± 90.2 vs 207.1 ± 84.7 mins, *p* = 0.002, respectively), whereas ablation time did not change significantly as the number of performed procedures increased (ANOVA *p* = 0.455) (Fig. 1).

**Fig. 2.**
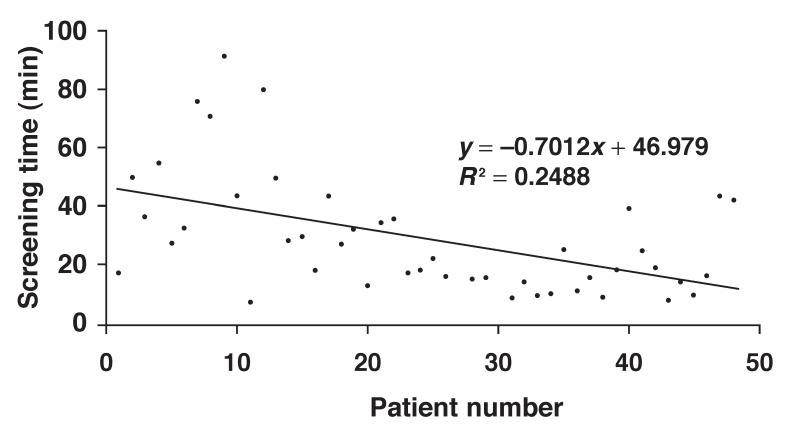
Scatter plot showing a decrescendo trend in screening time for the first half of the study group.

Fluoroscopy time showed a significant decrescendo trend for the first half of the patients, with an R^2^ of 0.25 and *p* = 0.006 (Fig. 2). There was no significant correlation for the second half of the procedures (*p* = 0.619).

## Complications

Eighty-six patients (91%) completed the postoperative in-hospital period and were discharged without any remarkable complication. There were four minor complications. One patient had a groin haematoma and one had meralgia paresthetica, both of which resolved spontaneously and were most likely related to the large 14F sheath used for Artisan™. One patient developed congestive heart failure 24 hours post procedure, which was related to the underlying structural heart disease and significant fluid infusion (at 17 ml/min) necessary at the time for cooling of the ablation catheter. Septicaemia was suspected in one case, which was probably co-incidental and was controlled with antibiotic therapy and supportive measures.

There were two intermediate-level complications. One patient developed a groin arteriovenous fistula, which was related to the large 14F sheath and was repaired surgically. One patient had late-onset pericardial effusion (on postoperative day 10), which was most likely related to fluid overload and inflammatory response to ablation and was treated successfully with pericardiocentesis.

There were three major complications. One patient had cardiac tamponade, treated immediately with pericardiocentesis. The patient was female and rather small in size. Another patient had left atrial perforation, which was repaired surgically. Similarly, the patient was female and rather small in size. This case was caused by ‘catheter snap’ during ablation on the upper aspect of the ridge between the appendage and left superior pulmonary vein (LSPV). Poor tissue quality/elasticity probably contributed to this complication, as the patient had rheumatoid arthritis and was on long-term methotrexate therapy.

One patient had aspiration pneumonia and blurring in the right eye due to embolus; his vision recovered after two months. This complication occurred because the patient was morbidly obese (160 kg) and it was difficult to keep his ACT above 250 s; he seemed resistant to unfractionated heparin. No case of pulmonary vein stenosis, transient ischaemic attack or stroke was reported throughout the post-procedure follow up.

## Follow up for relapse

**Fig. 3.**
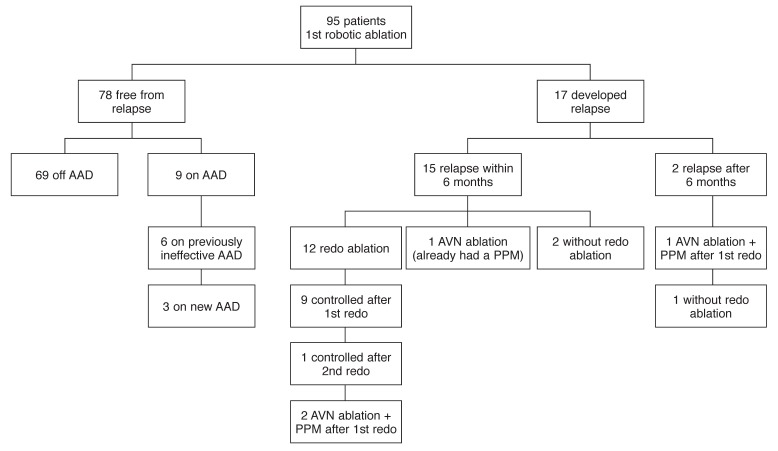
Flow chart showing outcomes for the whole study group; AAD, antiarrhythmic drugs; AV N, atrioventricular node; PPM, permanent pacemaker.

As of March 2011, patients were followed up for an average of 13.4 ± 3.6 months. A freedom from AF of 94.7% was achieved after an average of 1.15 attempts per patient. One patient refused to undergo more attempts after developing relapse, and four patients (4.2%) finally underwent AV node ablation with placement of a permanent pacemaker, one of which had developed late-onset relapse 13 months after robotically assisted ablation. Five patients (5.3%) underwent a redo ablation within the six-month follow up, seven patients (7.4%) had a redo procedure after six months, and one patient received two redo ablations at five and 10 months (Fig. 3).

The nine-month follow-up period was completed for 100% of patients; no cardiac mortality was observed among the study population within the nine-month period. However, one patient died of prostate cancer seven months post ablation. After one attempt, 26 patients (27.4%) were discharged from hospital off any sort of AADs.

At nine months’ follow up, 70 patients (73.7%) were free from arrhythmia off AADs, and 10 (10.5%) were AF-free on AADs, yielding a total freedom from AF of 84.2% without any redo procedures (Table 3). Considering those patients who relieved completely after a redo procedure within the nine months post ablation, freedom from relapse rose to 88.4%.

**Table 3.** First-Attempt Success Rates In Relation To The Type Of AF And Antiarrhythmic Drugs (AADs)

*Type of AF*	*Success off AADs n (%)*	*Success on AADs n (%)*	*Overall success n (%)*
Paroxysmal	12/12 (100)	1/1 (100)	13/13 (100)
Persistent	57/58 (98)	6/19 (32)	63/77 (82)
Long-standing persistent	0/0	2/5 (40)	2/5 (40)

Multivariate logistic regression analysis showed that longer-standing types of AF and ablation (radiofrequency) time are independent predictors of arrhythmia relapse within the nine-month period post ablation therapy (Table 4).

**Table 4.** Multivariate Logistic Regression Analysis Of AF Relapse Predictors After Robotic Ablation

*Variable*	*Odds Ratio*	*95% CI*
Age	0.9957	0.9386–1.0562
Concomitant flutter	0.8580	0.2334–3.1539
AF type	12.8330	1.4454–113.9412
Mapping system/ablation catheter	1.8701	0.4034–8.6688
Procedure time	0.9835	0.9700–0.9973
Radiofrequency time	1.0508	1.0104–1.0929
Screening time	1.0177	0.9838–1.0527

## Discussion

This is the first report on mid-term efficacy of robotically navigated catheter ablation in an unselected subset of patients with predominantly persistent AF. Overall success rate without any redos reported in this study is comparable to the results obtained in the largest randomised, controlled trial (*n* = 390 total; 197 robotic arm) performed to date by Di Biase *et al.* (84.2 v 85%).^11^ In the subset of patients with persistent AF, the results of this study compare favourably to that reported by Di Biase *et al.* (82 vs 70.9%).^11^ These results bolster our confidence in the robotic system as an efficacious treatment modality for patients with persistent AF.

The overall mid-term success rate of 88.4% after the 1.12 procedures per patient reported in this study is comparable to the results reported by Hlivak *et al.* (*n* = 69, success rate 86% after 1.2 procedures per patient).^13^ These studies consistently show that robotic ablation is also a clinically viable option for redo procedures in patients who do not respond to ablation on the first attempt.

Recently, an updated worldwide survey on the methods, efficacy and safety of manual catheter ablation for AF showed that across all surveyed centres, median overall success rates were 84.0% (79.7–88.6%; *n* = 9 590), 74.8% (66.1–80.0%; *n* = 4 712) and 71.0 (67.4–76.3%; *n* = 1 853) for paroxysmal, persistent and long-standing AF, respectively. For the main subset of patients in this study (i.e. patients with persistent AF), we see that overall success rate with the robotic ablation compared favourably with that of manual ablation. Given that robotic ablation technology, techniques and catheters are still in their infancy, the efficacy of this treatment modality has the potential to reliably surpass its manual counterpart in the near future.

As shown by the multivariate analysis, longer-standing types of AF and ablation (radiofrequency) time are independent predictors of arrhythmia relapse within the nine-month period post ablation therapy (Table 3). As described in the methods section, some of the more complex cases required ablation along the endocardial aspect of the coronary sinus or in an attempt to terminate the flutter or focal atrial tachycardia in addition to the standard PV isolation. Therefore, longer ablation time in fact points to a more complex underlying arrhythmia circuitry, which is the most likely explanation for its correlation with higher frequency of relapse. Association of long-standing AF type with higher relapse rate may also be related to the same aetiology.

In general, because the robotic navigation system significantly reduces physical operator strain, mitigates concerns over excessive radiation exposure during complex cases, and enables operators to perform complex ablation patterns regardless of catheter skills, operators are more likely to take on more complex and challenging AF cases. Hence, we expect to see longer ablation times as a surrogate for more complex cases associated with higher relapse rates in future studies.

As shown in Fig. 1, mean procedure and fluoroscopy times were statistically reduced after the first fifth of the patients (*n* = 19). Furthermore, fluoroscopy time showed a linear decrescendo trend for the first half of the patients (*n* = 48), after which point it reached a plateau. This observation was in line with the results reported by Di Biase *et al*., who showed statistically significant reduction in fluoroscopy time after the first 50 cases.^11^ These observations confirm that there is a learning curve in using the robotic navigation system and that operators can anticipate further reduction in fluoroscopy time, and hence safer operation once they overcome this learning curve. Reduction in procedure time allows for shorter cases, less physical operator strain and higher laboratory throughput.

Regarding major complications related to the robotic navigation system, specifically the incidence of cardiac tamponade and left atrial perforation, Hansen Medical’s new Lynx™ catheter, which is smaller in size (requires a 12F sheath) and more gentle (less rigid and lighter) than the Artisan™ catheter used in all the procedures reported herein, will probably reduce the risk of these complications, especially in small female patients with previous tissue quality/elasticity problems. Arteriovenous fistula, considered here as an intermediate-level complication, will likely occur less frequently with the new Lynx™ catheter that requires a 12F sheath as opposed to the 14F sheath size of Artisan™.

Additionally, the next generation of ablation catheters such as Carto SF (Biosense-Webster, Diamond Bar, CA, USA), which require a lower infusion rate (8 ml/min) due to design changes, producing more efficient cooling, will reduce the likelihood of post-procedural pericardial effusion or congestive heart failure. Lastly, the smaller size of the new Lynx catheter will significantly reduce the risk of minor complications such as groin haematoma or meralgia paresthetica.

It is important to note that despite our modified post-ablation anticoagulation regimen, which differs substantially from the international guidelines due to poor patient compliance with anticoagulation on warfarin and INR testing, no case of transient ischaemic attack or stroke was reported in the median nine-month post-ablation period. Physicians who care for similar types of patients in areas where compliance is an issue may find our modifications to the standard post-ablation anticoagulation regimen helpful.

The main limitations of this study are the fact that it lacked a matched control group with manual ablation and that there were relatively few subjects in the paroxysmal and long-standing persistent AF groups. Furthermore, some of the therapeutic modifications presented in this article, mainly the modified post-ablation anticoagulation regimen, were based on clinical experience and characteristics of our specific patient population and are not in conformity with international guidelines. Hence, we do not propose that our methods be adopted by other centres.

As a real-world study, we were treating symptomatic patients, with the main clinical endpoint being relief of symptoms. We were not able to confirm the absence of asymptomatic recurrence of AF without ECG Holter monitoring. Nevertheless, we believe that the safety and mid-term efficacy results of this study may provide valuable insights for the daily practice of medicine.

## Conclusion

The Sensei™ robotic navigation system offers a safe and effective approach for the treatment of AF. Its efficacy in patients with persistent AF is encouraging. Its overall success rate is comparable to manual techniques and impressive for a treatment approach that is relatively in its infancy. There is a learning curve with regard to fluoroscopy and procedure times, after which point reduction in radiation exposure and operator strain, as well as improvement in procedure throughputs are even more pronounced. Lynx™ as well as the newer generation of ablation catheters compatible with the Sensei™ system offer operators the possibility of even safer procedures with lower risks for complications.
